# Irrational prescription and its costs in neonatal surfactant therapy: public and private hospitals of Iran in 2018

**DOI:** 10.1186/s12887-023-04045-7

**Published:** 2023-05-20

**Authors:** Reyhane Izadi, Abbas Habibolahi, Nader Jahanmehr, Soheila Khodakarim

**Affiliations:** 1 Department of Health Care Management, School of Management and Information Sciences, Shiraz University of Medical Sciences, Shiraz, Iran; 2 Neonatal Health Department, Population, Family and School Health Office, Deputy of Health, Ministry of Health and Medical Education, Tehran, Iran; 3 Health Economics, Management and Policy Department, Virtual School of Medical Education & Management, Shahid Beheshti University of Medical Sciences, Tehran, Iran; 4 Prevention of Cardiovascular Disease Research Center, Shahid Beheshti University of Medical Sciences, Tehran, Iran; 5 Department of Biostatistics, School of Medicine, Shiraz University of Medical Sciences, Shiraz, Iran

**Keywords:** Irrational prescription, Costs, Neonate, Respiratory distress, Surfactant therapy, Respiratory distress

## Abstract

**Background:**

Irrational prescription and its subsequent costs are a major challenge worldwide. Health systems must provide appropriate conditions for the implementation of national and international strategies to prevent irrational prescription. The aim of the present study was to determine the irrational surfactant prescription among neonates with respiratory distress and the resulting direct medical costs for private and public hospitals in Iran.

**Methods:**

This was a cross-sectional descriptive study performed retrospectively using data belonged to 846 patients. Initially, the data were extracted from the patients’ medical records and the information system of the Ministry of Health. The obtained data were then compared with the surfactant prescription guideline. Afterward, each neonatal surfactant prescription was evaluated based on the three filters listed in the guideline (including right drug, right dose, and right time). Finally, chi-square and ANOVA tests were used to investigate the inter-variable relationships.

**Results:**

The results showed that 37.47% of the prescriptions were irrational and the average costs of each irrational prescription was calculated as 274.37 dollars. It was estimated that irrational prescriptions account for about 53% of the total surfactant prescription cost. Among the selected provinces, Tehran and Ahvaz had the worst and the best performance, respectively. As well, public hospitals outperformed private hospitals in terms of the in drug selection, but they underperformed them in terms of the right dose determination.

**Conclusion:**

The results of the present study are considered as a warning to insurance organizations, in order to reduce unnecessary costs caused by these irrational prescriptions by developing new service purchase protocols. Our suggestion is the use of educational interventions to reduce irrational prescriptions due to drug selection as well as using computer alert approaches to reduce irrational prescriptions caused by wrong dose administration.

## Background

Access to health services is one of the most important factors showing the medical progress in a country [1]. Although drugs play a key role in providing health care, they are expensive and account for a significant portion of total health expenditures [2]. In developing countries, a total of 25–70% of total health expenditures are spent on drugs, but only 10% of the total health expenditures are spent on drugs administration in high-income countries [3]. The high prices of drugs and lack of access to some important drugs due to economic fluctuations and sanctions are among the most important problems faced by Iranian in recent years [1]. Considering the recent political-economic challenges in Iran, now there is a need for proposing a targeted program and a sustainable method in order to prevent limited access to drugs in various situations and also to fund the pharmaceutical sector [4]. One of the factors affecting the drug-funding-sustainability and the efficient use of limited resources is the rational usage of drugs along with other health technologies [1].

World Health Organization (WHO) defined rational prescriptions as prescriptions in which the patient receives the right drug at the right dose and at the right time with the lowest possible cost, otherwise it is considered as irrational [5].

In this regard, numerous factors such as physician to population ratio [6, 7], payment methods, lack of prescription rules, nearly expired and expired drugs, and lack of appropriate medical guidelines, can lead to irrational prescriptions [8].

Irrational use of medicines poses a formidable challenge to health systems in many countries [2]. Drugs annually imposes 4 billion dollars to Iran’s health system, with patient’s out-of-pocket payment accounting for more than 45% of this estimation [1]. In this regard, irrational prescription imposes lots of financial pressure on insurers by increasing unnecessary medical expenses [9]. In addition, it could lead to economic challenges at the national level and place a heavy economic burden on health systems [10]. Tackling the issue of irrational drug prescribing is essential not only for optimal use of resources but also for improving healthcare delivery to ensure patient safety [2]. Irrational prescribing is a known preventable cause of adverse drug events and has an important impact on cost of care and public health indicators [11]. Although the correct prescription of drugs can help treat diseases, relieve symptoms, and reduce patients’ pain and suffering, their incorrect prescription can increase the risk of adverse drug reactions (ADRs), lead to treatment failures and hospitalizations, and cause significant morbidity and mortality [2, 10]; such side effects are three times higher in vulnerable pediatric populations [11]. Irrational prescription has been found as one of the most important challenges of the health system in Iran [12]. The results of a study have previously shown a doubled increase in prescription of some drugs in Iran in the last decade [1]. Correspondingly, numerous international studies have also referred to the induced demand and irrational prescription in laboratory and imaging services, frequent medical visits [13], cesarean Sect. [14], and medication and neonatal services [14, 15].

To reduce these irrational prescriptions, 12 key strategies has been proposed by WHO for health care administrators. These strategies include the establishment of a multidisciplinary national body to coordinate policies, formulating clinical guidelines, selecting based on the list of essential drugs, setting up drug and therapeutics committees, promoting problem-based training in pharmacotherapy, continuing in-service medical education, promoting the supervision system, providing independent information, promoting public education about medicines, applying regulations, reserving governmental expenditure to equitable availability of drugs, eliminating perverse financial incentives [5].

In recent years, according to unpublished data from the Ministry of Health of Iran as well as the interviews with policy makers in the field of neonatal health, the surfactant prescription in neonates is rapidly growing. Surfactant, which is used to treat respiratory distress diseases among neonates, is one of the vital and expensive drugs in Iranian pharmacopoeia. Respiratory distress diseases are known as one of the leading causes of neonatal death in developing countries [16], among which respiratory distress syndrome (NRDS) is the most common one [17]. This syndrome is a pulmonary disorder caused by pulmonary surfactant deficiency [18], which may possibly lead to pulmonary air leak, intracranial hemorrhage, and eventual neonatal death [19]. The results of a previous retrospective study conducted in Iran showed that 65.6% of neonates under 34 weeks of gestation age have this syndrome [20]. Surfactant therapy has revolutionized the treatment of neonatal respiratory failure in recent decades. Currently, there are various artificial and natural surfactants in the commercial health market. However, there are only four natural surfactants in the Iranian health market, as follows: Alveofact, Survanta, BLES^1^, and Curosurf. Of note, there was an increase in the induced demand in Iran since the implementation of Health System Reform Plan (HSRP) in 2014 and the increased tariffs [21].

According to the directors of the Neonatal Health Department of the Ministry of Health of Iran, it is believed that the increase in surfactant prescription in recent years may possibly be due to irrational prescription and induced demand as the results of the implementation of HSRP. In recent years, many studies have been conducted on investigating various aspects of neonatal respiratory distress as well as the effects of various types of surfactants on it [19, 20]. As well, several studies have previously investigated the irrational prescription of many drugs and drug costs [14, 15, 22]. However, there has been no study in Iran performed on the irrational prescription of surfactant in the treatment of neonatal respiratory distress so far. The present study was conducted at the request of the ministry of health of Iran and with the aim of determining the rate of the irrational surfactant prescription in the treatment of neonatal respiratory distress in public and private hospitals as well as calculating the direct medical costs.

## Methods

### Study design

A cross-sectional study was conducted in public and private hospitals in Iran during 2018 with the aim of determining the amount of irrational prescription and the resulting direct medical costs.

### Population and sampling

**Fig. 1:**
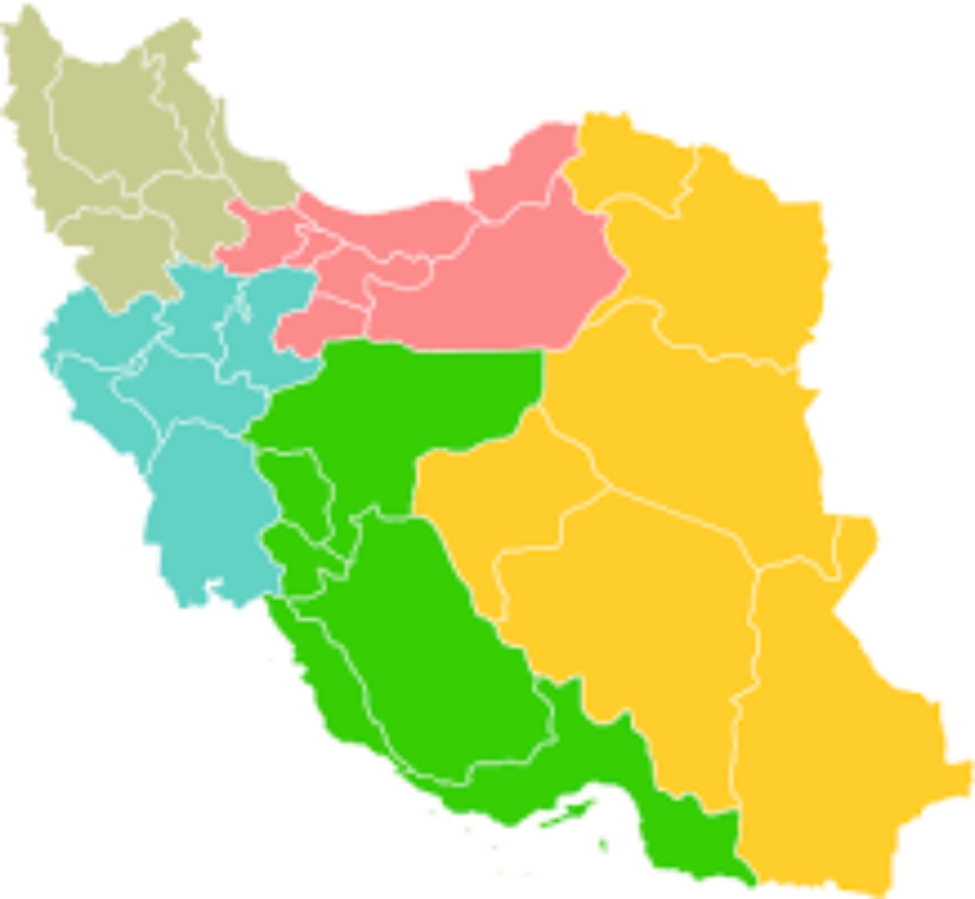
Five divisions of national regions in Iran

The study population included all Iranian neonates who underwent surfactant therapy in 2018. In the present study, the cluster-stratified sampling method was used for selecting the cases. First, the provinces of Iran were divided into five main regions according to the classification of the Ministry of Interior (Fig. (1)). Second, a province with the highest number of neonatal surfactant prescriptions was selected from each region based on the information system of the Ministry of Health. Moreover, because this is a subspecialty service and may not be provided in all cities, only provincial centers were included in the current study, and this was done using purposeful sampling. Next, a private hospital along with a public hospital with the highest number of surfactant prescriptions were selected from each provincial center. Finally, based on the available list of neonates in IMAN Net, the sample was randomly selected. The list of medical records belonged to the selected neonates were then sent to the hospitals and the hospitals were asked to send the medical records to the Ministry of Health within a certain period of time. In the present study, the sample size included 784 cases of surfactant prescriptions, considering p = 0.5, $\alpha$ = 0.05, 05 = 0.20, and d = 0.05. However, the number of the prescriptions reached 846 cases in proportion to the number of the samples specified for each hospital, in order to increase the reliability of the obtained results.

### Data collection and analysis

In order to determine the rate of irrational prescription, the National Guideline for Surfactant Prescription among Iranian Neonates (2018), which was firstly developed by the Ministry of Health and Medical Education, was used as the instrument in the present study. The required data were extracted from the Iranian Maternal and Neonatal Network (IMAN Net) and the medical records in private and public hospitals.

Based on this guideline, three steps of right drug, right dose, and the right time for a rational prescription were considered, and in this study, each one of the cases of surfactant prescription was investigated based on these three filters, respectively.

#### Right drug

Based on the prescribed indications, was surfactant drug a right decision for the neonates?

#### Right dose

Based on the surfactant type and the neonate weight, was the right dose prescribed for the neonate?

#### Right time

Was the surfactant injected at the right time to the neonate?

#### Total index of prescription

This index is a combination of the previous three components, which can be either in a verifiable (no missing data) or in an unverifiable (missing data) status. Moreover, the verifiable status of this index can be either rational or irrational, which were presented as the indexes of rational prescribing (IRP) and irrational prescribing (IIP) in the current study, respectively.

#### Index of rational prescribing (IRP)

When all three components of this index are rational, it means that all three components of drug selection, dose, and time are rational as well.

#### Index of irrational prescribing (IIP)

When at least one of the three components of this index is irrational, it means that either the drug selection, dose or time is irrational.

### Part 1: measurement of irrational prescription

#### 1- Right drug

In the first step, we determined whether the patient was clinically eligible to undergo surfactant replacement therapy based on the four indications stated in the national guideline (Table 1). Thereafter, each neonatal surfactant prescription was evaluated based on all these four indications. Accordingly, this step had the following two parts.

- Each indication consists of two variables, and an indication for one prescription was considered as rational only if both variables were rational. As well, if there was only one irrational variable, the related indication was considered as irrational for that prescription. Additionally, an indication was considered as unverifiable when both variables were unverifiable or one of the variables was unverifiable and the other one was rational. Afterward, by juxtaposing the variables of an indication, we determined the state of each indication for a specific prescription (either rational, irrational, or unverifiable), apart from the status of the other indications.
- Next, by simultaneously evaluating all these four indications, we determined the number of patients who had irrational surfactant selection. The surfactant selection was considered as irrational when all the four indications emphasized the irrationality of a prescription. Moreover, the surfactant selection was considered as rational when at least one indication has confirmed the rationality of that prescription. Besides, other cases in which the combination of these indications was in an irrational, unverifiable state, they were all considered as unverifiable.

**Table 1:** Indications of surfactant prescription in neonates

Prescription indications	Indication variables	Status of variables	Define the status of variables
Indication 1	Variable a: Gestational Age	Rational	≥ 37 weeks
		Irrational	< 37 weeks
	Variable b: Progress in the resuscitation operations in the operating / delivery room	Rational	Neonate needs intubation-based resuscitation in the operating/delivery room
		Irrational	Neonate needs no intubation-based resuscitation in the operating /delivery room
Indication 2	Variable a: Gestational Age	Rational	≥ 37 weeks
		Irrational	< 37 weeks
	Variable b: Type of respiratory support before surfactant prescription and Status of the need for the increased CPAP and FIO_2_^a^	Rational	NCPAP and need to increase CPAP ≥ 8 cm/H_2_O or FIO_2_ ≥ 30%
		Irrational	Not- NCPAP
Indication 3	Variable a: Gestational Age	Rational	≥ 37 weeks
		Irrational	< 37 weeks
	Variable b: Chest radiography in the first 48 h of birth	Rational	Abnormal
		Irrational	Normal
Indication 4	Variable a: Type of respiratory distress disease	Rational	RDS or MAS ^b^or PNA ^c^
		Irrational	not-RDS & not-MAS & not-PNA
	Variable b: Progress in the resuscitation operations	Rational	Use of endotracheal intubation
		Irrational	using no endotracheal intubation

^a^ Fraction of inspired oxygen

^b^ Meconium aspiration syndrome

^c^ Intrauterine pneumonia

#### 2- Right dose

In the second step, a “standard dose” was established based on the type of the prescribed surfactant and the neonate’s weight for each patient. This standard dose was then compared with the prescribed dose. If the dose of the three surfactants of Alveofact, BLES, and Survanta deviated the standard dose by a maximum of ± 0.50, the prescribed dose was considered as irrational in the present study as well. Since the minimum and maximum administered doses of Curosurf surfactant are provided in the guideline, the minimum and maximum standard doses were determined for each neonate. In addition, if the prescribed dose was in the “standard dose range” or even if it deviated the standard dose range up to two decimal digits, it was still considered as rational. These doses, which were regarded as irrational in terms of the standard stated in the national guideline, by the suggestion of the research team were considered as rational, which are listed as “rational doses without sensitivity " in the findings. Finally, if the data on the dose was missing, this component would be considered as unverifiable.

#### 3- Right time

In this step, it was determined whether the patient received the surfactant at the right time. In terms of the above-mentioned guideline, we considered the time periods less and more than 48 h of birth as rational and irrational, respectively.

### Part 2: measurement of the direct medical costs^2^

In this section, two types of the fixed and variable costs were identified for each prescription. Accordingly, the fixed cost includes medical consumables (including chip tube, NGtube, syringe, gloves, etc.), which are the same for all types of prescriptions. Variable cost includes the price of surfactant vial in 2018, which was calculated to be varied according to the type of the prescribed surfactant. Accordingly, the related prices were determined based on the average price of the largest surfactant distributor company in Iran in 2018.

### Statistical analysis

In the present study, data analysis was conducted using Stata ver. 19. Quantitative variables were reported by mean and standard deviation, and qualitative variables were also reported by frequency and percentage. Finally, chi-square and ANOVA tests were used to investigate the inter-variable relationships.

## Results^3^

### Total index of prescribing

The total index of the prescribing and its sub-components is listed in Table 2:

**Table 2:** The total index of the drug prescribing and its components

Status prescription		Drug selection	Drug dose	Prescription time	The total index of drug prescribing
Verifiable	Rational	709 (83.80)	360 (42.55)	804 (95.03)	278 (32.86)
	Irrational	121 (14.30)	232 (27.42)	9 (1.06)	317 (37.47)
Unverifiable		16 (1.89)	254 (30.02)	33 (3.90)	251 (29.66)

*Data are presented as number (%)

### Total index of the prescription in both public and private hospitals

The results show some significant differences between public and private centers in terms of the total index of prescribing, especially regarding the drug selection component (Table 3).

**Table 3:** The total index of drug prescribing and its components in both private and public hospitals

The total index of drug prescribing and its components	Type of hospital	Status verification							Number (%)	P-value	
Drug selection	Public	Verifiable			Rational				606 (92.37)	< 0.001	
					Irrational				50 (7.62)		
		Unverifiable							12		
	Private	Verifiable	Rational						103 (59.19)		
			Irrational						71 (40.80)		
		Unverifiable							4		
^a^ Drug dose	Public	Verifiable			Rational				261 (59.58)	0.304	
					Irrational				177 (40.40)		
		Unverifiable							230		
	Private	Verifiable		Rational					99 (64.28)		
				Irrational					55 (35.71)		
		Unverifiable							24		
Drug time	Public	Verifiable				Rational			631 (98.59)	0.202	
						Irrational			9 (1.40)		
		Unverifiable							28		
	Private	Verifiable				Rational			173 (100)		
						Irrational			0 (0)		
		Unverifiable							5		
The total index of drug prescribing	Public	Verifiable						Rational	223 (51.26)	< 0.001	
								Irrational	212 (48.73)		
		Unverifiable							233		
	Private	Verifiable					Rational		55 (34.37)		
							Irrational		105 (65.62)		
		Unverifiable							18		

Data are presented as number and number (%)

^a^ Considering the unverifiable items in the “Drug dose” Sig < 0.001

### Total index of the prescribing in provinces of Iran

In Iran, according to IMAN Net 13% of all surfactant prescriptions in neonates is related to Tehran province. It was also found that the total index of prescription had the worst and best statuses in Tehran and Ahvaz provinces, respectively (Table 4).

**Table 4:** Total index of the prescribing based on the selected provinces

Selected province	Total surfactant prescription	Number of reviewed prescriptions	Drug selection + Drug dose + Prescription time		
			Verifiable prescriptions ^a^	Number (%)	Unverifiable prescriptions
Tehran	(13.00)1468	232 (27.42)	190 (81.89)		42 (18.10)
			The total index of the rational prescribing	62 (32.63)	
			The total index of the irrational prescribing	128 (67.36)	
Fars	(7.5)857	133 (15.72)	104 (78.19)		29 (21.80)
			The total index of the rational prescription	53 (50.96)	
			The total index of the irrational prescribing	51 (49.03)	
East Azerbaijan	(8.48)957	197 (23.28)	138 (70.05)		59 (29.94)
			The total index of the rational prescribing	77 (55.79)	
			The total index of the irrational prescribing	61 (44.20)	
Ahvaz	(7.31)825	167 (19.73)	76 (45.50)		91 (54.49)
			The total index of the rational prescribing	46 (60.53)	
			The total index of the irrational prescribing	30 (39.47)	
Razavi Khorasan	(4.32)488	117 (13.82)	87 (74.35)		30 (25.64)
			The total index of the rational prescribing	40 (45.97)	
			The total index of the irrational prescribing	47 (54.02)	
Total ^b^ in these five province	4595(40.61)	846(100)	595 (70.33)		251 (29.66)
			The total index of the rational prescribing	278(46.72)	
			The total index of the irrational prescribing	317(53.27)	

^a^ Data are presented as number (%) and the percentages written in column 5 are based on the verifiable prescriptions

^b^ Total number of surfactant prescriptions in Iran was calculated as 11,284

### Direct medical costs

The cost of one surfactant prescription depending on the type of surfactant varies from a minimum of 298.12 dollars (Curosurf) to a maximum of 385.80 dollars (Alveofact) in Iran (2018).

A prescription was considered as irrational either when the drug selection or dose or time was irrational or when more than one of these factors were irrational. Table 5 lists the reason, number, and costs of irrational prescription:

**Table 5:** Frequency and costs of irrational prescriptions based on different reasons

Type of surfactant	Irrational drug selection or irrational drug selection and dose	Only irrational dose	proportionally added unverifiable dose items ^c^	A total number of irrational prescriptions	Rational doses with no sensitivity	A total number of irrational prescriptions + rational doses with no sensitivity
Curosurf	86	100	51	237	18	254
	$25,638.62	$29,812.35	$15,204.30	$70,655.28	$5,366.22	$76,021.51
Survanta	23	70	35	128	57	183
	$5,714.03	$17,390.54	$8,695.27	$31,799.84	$14,160.86	$45,712.28
BLES	14	16	8	38	29	67
	$2,659.72	$3,039.69	$1,519.84	$7,219.26	$7,204.65	$14,423.92
Alveofact	1	2	1	4	16	20
	$385.80	$771.61	$385.80	$1,543.22	$6,172.91	$7,716.13
Total	124	188	95	407	120	527
	$34,398.19	$51,014.20	$25,805.22	$111,217.63	$32,904.65	$143,873.85

Data are presented as number and cost $

^a^ One case in which the type of surfactant was not known, but it was irrational in the drug selection (Column 2) and two cases that were not unverifiable in general (Column 4) were all added to the Curosurf category.

^b^ One case of unverifiable prescriptions was proportionally added to Survanta.

^c^ There were five prescriptions in which the drug selection and dose were rational, but the drug time was irrational. These five cases are shown in Column 4.

The average cost of each wrong drug selection was $34,398.19/124 = $277.40

The average cost of each wrong dose was $51,014.20/188 = $271.35

The average cost of each irrational prescription was $274.37

In Iran, a total of 11,284 neonates with respiratory distress disease underwent surfactant treatment. According to the results of the present study, it was estimated that in private and public hospitals, there were 1991 and 4021 irrational prescriptions in total, respectively. According to the average price of each type of surfactant prescription, the total direct medical cost of surfactant prescriptions for neonates with respiratory distress was estimated as 3,096,087.32 dollars and the direct medical cost due to irrational prescription in these neonates was obtained as 1,649,563.71 dollars. Approximately, 53% of these costs were related to irrational prescriptions. Notably, by taking the costs of Relative Value Units of surfactant prescription (RVUs) into account, the costs of irrational prescriptions reached 2,077,207.18 dollars (Table 6).

**Table 6:** Direct medical costs and RVUs due to surfactant prescription among neonates

Total neonates ^a^	Total direct medical costs	Rational prescriptions	Irrational prescriptions ^b^		RVU for irrational prescriptions ^c^		DMC ^d^ due to irrational prescriptions ^e^	DMC and RVU for irrational prescriptions
			Public	Private	Public	Private		
11,284	$3,096,087.32	5272	4021	1991	$201,390.82	$226,252.64	$1,649,563.71	$2,077,207.18

^a^ provided based on the information system of the Ministry of Health

^b^ These values were calculated in proportion to the research results (among 595 verifiable cases, 317 were irrational cases (private hospital = 105, public hospital = 212) and 278 were rational (hospital = 55, public hospital = 223))

^c^ The cost of RVUs for surfactant-replacement therapy in neonates in Iran (2018) was calculated as 2.78 dollars in public hospitals and as 6.31 dollars in private hospitals and the service code for surfactant prescription was 18 RVUs. (Thus, the surfactant service code was 50.08 and 113.63 dollars for public hospitals and private hospitals, respectively)

^d^ Direct medical costs

^e^ This cost was obtained by taking the average cost of 407 irrational items into account from Table 5

## Discussion

### Total index of prescribing (drug selection + drug dose + drug time)

The main aim of the present study was determining the rate of irrational surfactant prescription among Iranian neonates with respiratory distress disease based on the national guideline. The results show that the total prescription index was rational for 278 (46.72%) neonates and irrational for 317 (53.27%) neonates. In a similar study on 4182 prescriptions, Campino et al. (2009) has previously showed that the rate of prescription error in the NICU is 20.70% [23]. Moreover, Machado et al. (2015) conducted a study to measure the rate of prescription error in the NICU and as a result showed that this rate was 43.50% and respiratory drugs were ranked as the fourth in terms of error [24].

Our suggestion to reduce such errors is to use educational interventions because these interventions to a large extent can lead to some changes in physicians’ prescription behavior [25]. As well, creating supportive attitudes to control the induced demand in health system managers can be effective on reducing the rate of irrational prescriptions [9].

### Total index of prescribing in private and public hospitals

In Iran, the private and public sectors both provide health care and treatment services; however, the public sector and specially the Ministry of Health play a more significant role in this regard [26]. The public sector provides all health care in all regions of the country [27]; almost more than 90% of health services are provided by the government [28]. The private sector is mainly focused on providing secondary and tertiary health care in urban areas [27]. In this country, a national system of medical tariff setting for provider reimbursement is used since 1972, and one of the most important challenges of the tariff setting process is that there is a significant difference in medical tariffs for similar services between the public and private sectors. This difference led to a substantial income discrepancy and is one of the main motivating factors for working in the private sector and developing dual practices, and even increasing irrational prescriptions in the private sector [29].

The results of this study show that the total index of prescribing, especially the component of drug selection, was significantly different between public and private centers. The drug selection was found to be irrational in 7.62% of the verifiable prescriptions in public hospitals, while this rate was 40.80% in private hospitals. Public hospitals seem to have more commitment to rational prescription indications. Other researchers have also confirmed that patients receive better treatment services in public centers compared to private ones according to clinical guidelines. In fact, since in the public sector, the educational level is better and the opportunities for training are more, therefore, higher rates of correct diagnosis and correct prescription are provided; also, this difference in the quality of care between the public and private sectors likely reflects financial incentive aspects [30]. Of note, most of the changes made by physicians in the volume of services are related to financial incentives [31]. Considering that RVUs of surfactant prescription in the Iranian private sector was more than twice the public sector (for some other services such as internal medicine, anesthesia and surgery services, in the private sector up to 10 times more than the public sector), the financial incentives of physicians can be considered as a factor leading to an increase in the irrational prescription due to wrong drug selection in private sectors [29].

In the present study, the index of irrational drug prescribing in public and private hospitals was obtained as 48.73% and 65.63%, respectively. Although the percentage of right drug selection was higher in public hospitals, they had lower percentage of right dose compared to private hospitals. Aronson et al. have stated that one of the most important cause of human errors in physicians is fatigue. Fatigue is known as one of the most important human causes of error among physicians [32]. Since the results of our research show that the number of surfactant prescriptions in public hospitals is about four times more than that of private hospitals, it can be said that both overwork and fatigue of physicians in the public sector are effective factors on reducing their accuracy in calculating weighed-based dose [32]. On the other hand, performing regular monitoring programs in the private sector leads to a reduction in drug prescription error compared to the public sector [8]. In line with our results, Bashir et al. (2018) in their study referred to 37% irrational prescriptions in private clinics as well as 74.5% in public centers [33]. Furthermore, Bassoum et al. (2018) found the performance of none of the public and private centers in consistent with the index of rational drug prescribing [34].

### Total index of prescribing in the provinces of Iran

The total index of irrational drug prescribing was the highest in Tehran province (67.36%) and the lowest one was in Ahvaz province (39.47%). The results show that Tehran province has the weakest performance in surfactant prescription in neonates among the selected provinces that were investigated in the current study.

The situation of organizations also is effective on the occurrence of errors. Although human factors often are the first cause of error, and the environment as a latent factor has a significant impact on the occurrence of error. It is expected that the higher the number of prescriptions, the higher the probability of the occurrence of error[35]. Considering that 13% of all surfactant prescriptions in Iran are related to Tehran province, so it was expected that the occurrence of error would be higher in this province as well. In other words, more prescriptions, as a latent factor, have been effective on causing more errors in Tehran. On the other hand, it was shown that the more complex the steps of the prescription process, the higher the probability of the occurrence of error [35]. Many neonates admitted to hospitals in Tehran province (capital of Iran) are patients referred from other provinces, because they were not receiving effective treatments in other provinces. These patients probably had a more complex treatment process, which is potentially considered as a latent factor that had consequently led to an increase in prescription errors [35]. Other evidence also shows that some patients may be advised to travel to Tehran for their treatment and to receive higher-quality care because advanced treatment facilities are not available in their place of residence [36].

### Direct medical costs due to irrational surfactant prescription

The direct cost of treatment due to irrational prescription surfactant among the selected hospitals of the present study was calculated as 111,217.63 dollars. Besides, the average cost of each irrational surfactant prescription was 274.37 dollars. It was estimated that the direct cost of treatment for all Iranian neonates with respiratory distress disease who underwent surfactant treatment was 3,096,087.32 dollars, of whom about 53% was due to irrational prescriptions.

In this regard, the results of a study conducted in Nepal revealed that 20–52% of the total drug costs are due to irrational drug prescriptions and the average drug cost per prescription was calculated as 5.7 Nepalese rupees [37]. As well, a study in Ghana showed that if prescriptions were based on the recommendations by national health officials, drug costs would drop by 70% [38].

The cost of irrational prescriptions is considered as a barrier that deprives people of health services [37] and challenges the issue of health equity [9]. ​​In low-income countries, drug costs are one of the reasons of poverty in families [39]. Since hospitals have the opportunity to use the income obtained from drug prescription to compensate for medical deductions, this may encourage them to increase revenue by prescribing more drugs, and consequently expensive drugs are preferred by hospitals [40]. The Islamic Republic of Iran, with an average annual growth of 11.50% in the period from 2000 to 2008, compared to the average growth of 7% in developing countries and an average growth of 9% globally, is one of the countries with the highest drug prescription rate [1, 41]. A study showed that when physicians’ income decreases due to the increased In Iran, the private number of physicians in society, they compensate for this decreased income level by increasing their unnecessary demands from their patients [42]. Considering the available findings and evidence in the present study, it is clear that a part of the prescription costs is due to irrational prescriptions imposed on society. In Iran, it may be possible to control, to some extent, the induced demand and irrational prescription by improving managers’ insights on supporting induced demand control, and correcting payment methods and referral systems [9].

One of the main limitations of the present study was that, according to national standards, we expected medical records to be available electronically in hospitals. However, this was not the case in some of the hospitals, and the research team had to wait for the medical records to be scanned and sent; therefore, the study period was longer than we had anticipated. In addition, the quality of medical records may have been different in the investigated hospitals, which could affect the findings. Another limitation of this study is that the average length of stay (LOS) was not considered in the analysis, whereas a longer LOS can increase the possibility of any medication error. It should be noted that this research is based on a descriptive design and few controls have been used to identify statistical differences between regions. Also, in this research, without investigating the root causes of these irrational prescriptions, we have only determined the amount of these prescriptions; for future research, we strongly recommend investigating the root causes of these irrational prescriptions.

## Conclusion

The present study aimed to determine the irrational surfactant prescription among neonates and then to compare this index in private and public hospitals of the selected provinces in Iran. The results show that Tehran, which is the capital of Iran, has the worst performance in terms of surfactant prescription among the selected provinces. It was also found that although the percentage of right drug selection was higher in public hospitals, they had lower percentage of right dose compared to private hospitals. Finally, it was estimated that about 53% of the total cost of surfactant therapy in neonates was related to irrational prescriptions.

It is suggested to form multidisciplinary specialized committees consisting of different stakeholders at the regional level, in order to evaluate the performance of medical centers more effectively, and formulate and implement appropriate policies and guidelines. It must be acknowledged that we can all make errors in any organization. Overall, several reasons exist for these errors and different solutions to prevent them. However, we must begin by recognizing that errors are undeniable and then take some steps to minimize them. The first step in reducing errors is identifying their extent. Thereafter, the root causes of the errors must be identified, and finally, preventive strategies must be applied. Considering the fact that surfactant is one of the most expensive drugs in Iranian Pharmacopoeia, the results of the present study are regarded as a necessity and a warning for insurance organizations to develop new protocols for purchasing services during the process of surfactant therapy in neonates, in order to prevent unnecessary burdens caused by the irrational prescription of this drug.

## Data Availability

The datasets generated and/or analysed during the current study are not publicly available due to concerns and laws related to information confidentiality, but are available from the corresponding author on reasonable request.

## Footnotes

1 Bovine Lipid Extract Surfactant.

2 According to the statistics of the Central Bank of Iran, the price of United States dollar (USD) in Iran in the study year (2018) has varied from a minimum of 32,415 rials to a maximum of 37,695 rials. In the present study, the average USD price (as 34,214 rials) was considered

3 Significance of the results of the present study was assessed after excluding the unverifiable cases

## Abbreviations

HSRP: Health System Reform Plan
IIP: Index of Irrational Prescribing
IMAN Net: Iranian Maternal and Neonatal Network
IRP: Index of Rational Prescribing
RVUs: Relative Value Units
WHO: World Health Organization
DMC: Direct Medical Cost
ADRs: Adverse Drug Reactions
LOS: Average Length of Stay.
